# Starting A New Robotic Surgery Program for Mitral Valve Repair. Lessons Learned from The First Nine Months

**DOI:** 10.3390/jcm10225439

**Published:** 2021-11-21

**Authors:** Antonio Piperata, Olivier Busuttil, Nicolas d’Ostrevy, Jean-Luc Jansens, Saud Taymoor, Besart Cuko, Thomas Modine, Mathieu Pernot, Louis Labrousse

**Affiliations:** 1 Department of Cardiology and Cardio-Vascular Surgery, Hopital Cardiologique de Haut-Leveque, Bordeaux University Hospital, 33604 Bordeaux, France; olivier.busuttil@chu-bordeaux.fr (O.B.); nicolasdostrevy@gmail.com (N.d.); drsaud6@gmail.com (S.T.); drcukobesart@gmail.com (B.C.); thomasmodine@gmail.com (T.M.); mathieu.pernot@chu-bordeaux.fr (M.P.); louis.labrousse@chu-bordeaux.fr (L.L.); 2 Department of Cardiac Surgery, Erasme Hospital of Brussels, ULB, Belgium free University of Brussels (ULB), Hôpital Erasme, 1070 Brussels, Belgium; jl.jansens@me.com

**Keywords:** robotic mitral valve repair, heart valve surgery, surgical techniques

## Abstract

(1) Background: Although transcatheter technology is rapidly growing and represents a promising strategy, the surgical approach remains the best way to repair a degenerative mitral valve regurgitation. In this context, robotic surgery is technologically the most advanced method of minimally invasive mitral valve repair. The aim of this study is to present the preliminary results of the initial single-center experience with a new robotic mitral valve repair program. (2) Methods: We retrospectively reviewed the records of patients who underwent robotic mitral valve repair at our Institution between January and September 2021. (3) Results: A total of 29 patients underwent mitral valve repair with annuloplasty and chordal implantation to treat degenerative mitral regurgitation. The procedure’s success was achieved in 97% of patients. The 30-day cardiac-related mortality was 0%. The median CPB and cross-clamp times were 189 and 111 min, respectively, with a progressive reduction from the beginning of the robotic program. (4) Conclusions: Considering all the limitations related to the small sample, the presented results of robotic mitral valve repair appear to be encouraging and acceptable. A careful patient selection, a dedicated team, and a robust experience in surgical mitral valve repair are the fundamentals to start a new robotic mitral surgery program.

## 1. Introduction

The concept of minimally invasive cardiac surgery has dramatically changed clinical practice over the past two decades with the final goal to reduce invasiveness, post-operative pain, complications, and hospital stays [1,2]. Although transcatheter technology is rapidly growing and represents a promising strategy for valve replacement [3,4], the surgical approach remains the best technique to repair a degenerative mitral valve regurgitation [5].

In this context, robotic surgery is technologically the most advanced method of minimally invasive mitral valve repair. However, the worldwide spread of this approach is held back for several reasons, including the high costs and an apparently long learning curve [6,7,8].

In this study, we aim to present the results of our initial single-center experience with the DaVinci system (DaVinci X surgical system, Intuitive Surgical Inc., Sunnyvale, CA, USA) for mitral valve repair, sharing the highlights of our program. A special focus is also dedicated to our “respect technique” and to the encouraging learning curve achieved, despite the lack of a minimally invasive surgery program so far at our Institution.

## 2. Materials and Methods

Between January and September 2021, 44 patients underwent cardiac surgery procedures using the DaVinci system at the University Hospital of Bordeaux (France). Table 1 shows the overall population and the procedures performed.

A total of 32 patients underwent mitral valve surgery. Three of them underwent mitral valve replacement as their first intention, and for this reason, they were excluded from the study. The remaining 29 patients underwent a mitral repair for degenerative mitral valve regurgitation and were included in this retrospective single-center study. The primary indication for surgery included severe mitral regurgitation with posterior and/or anterior leaflet prolapse, Barlow disease, and fibroelastoma. The exclusion criteria included the presence of severe obesity and patients previously treated with cardiac surgery or radiotherapy. Other possible contraindications were the evidence of thoracic or vascular malformations observed on a pre-operative CT scan. All procedures were performed by 1 surgeon (L.L.) with the patients under mildly hypothermic cardiopulmonary bypass (CPB). Post-operative outcomes were defined as occurring within the 30th post-operative day. Pre- and post-operative echocardiographic evaluations were performed according to current recommendations [9] by board-certified cardiologists, and mitral valve regurgitation was classified as follows: effective regurgitant orifice area (EROA), <20 mm^2^ and/or, regurgitant volume (RVol) < 30 mL, mild-to-moderate (2+/4+) (EROA 20–29 mm^2^ and RVol 30–39 mL); moderate (3+/4+) (EROA 30–44 mm^2^, RVol 45–59 mL); or severe (4+/4+) (EROA > 40 mm^2^, RVol > 60 mL). The analysis of the surgical outcomes was performed by reviewing electronic health records.

### Operative Setting and Procedure

All patients underwent pre-operative multislice computer tomography (CT) to assess the vessels’ anatomy and calcifications and to identify an orthogonal trajectory to the mitral valve annular plane to facilitate the procedure.

**Figure 1:**
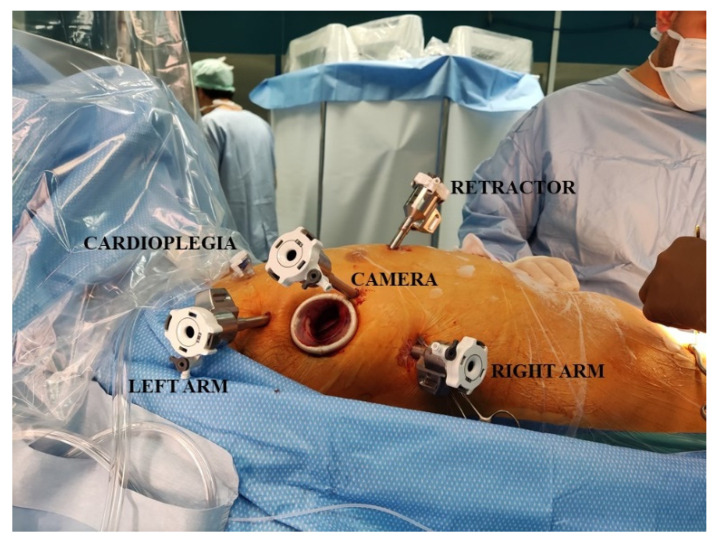
This picture shows the operative setting and the insertion sites of the trocars.

After double-lumen intubation, a Swan-Ganz catheter, right internal jugular venous line, and a 3D transoesophageal echocardiography (TOE) probe were inserted. The patient’s right side was elevated 30 degrees from the horizontal plane to obtain the best orthogonal access plane in front of the mitral valve (Figure 1).

After systemic heparinization, the cardio-pulmonary bypass was started according to the standard femoral cannulation technique, and the patient was cooled to 34 °C. Additional bicaval venous cannulation was performed via the right femoral and jugular veins if the right-side procedure was anticipated. A 3 cm incision at the level of the fourth intercostal space (ICS) was made along the anterior axillary line. Trocars were placed in the third and fifth ICS, and a camera trocar was placed anterior to the working port in the fourth ICS according to the pre-operative CT scan evaluation (Figure 1). A trocar was inserted in the submammary fourth/fifth ICS for insertion of the dynamic mitral retractor. The DaVinci system was then placed on the left side of the patient, and robotic instruments, 3D camera, and retractor were inserted in the thorax. The first surgeon was placed on the principal console, the assistant on the right side of the patients, and an additional assistant on the second console.

The video shows the operative setting and the whole procedure.

The pericardium was gently opened 2 cm anterior to the phrenic nerve and gently retracted with two stitches. An antegrade cardioplegia needle/vent was placed in the ascending aorta. A trans-thoracic aortic clamp was passed through the thorax wall in the second or third ICS in the middle axillary line and positioned around the aorta allowing the aortic clamp and cardioplegic arrest with the injection of 2L of Bretschneider cold cardioplegia (Custodiol HTK Solution; Essential Pharmaceuticals, LLC, Ewing, NJ, USA)

The left atrium was opened, and the mitral valve exposure was completed through the robotic retractor. A meticulous analysis of the mitral valve is mandatory before starting the robotic mitral valve repair. This step includes the leaflets evaluation, coaptation height, the presence of clefts, and other possible valve injuries. Our approach for treating mitral valve regurgitation does not involve leaflet resection. This technique is performed with the placement of artificial chordae (4-0 expanded polytetrafluoroethylene) between the anterior and posterior papillary muscles, and the free margin of the prolapsing leaflet allows correcting the mitral regurgitation respecting the valve.

**Figure 2:**
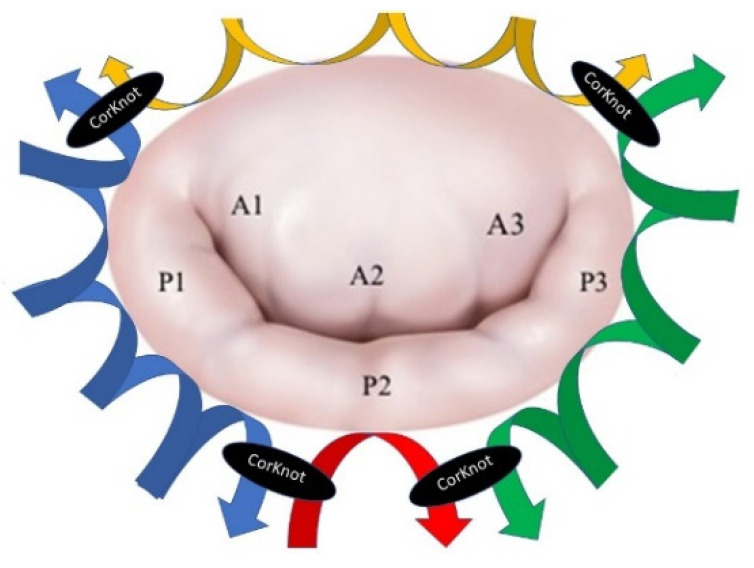
The annuloplasty technique.

The procedure was then completed with the annuloplasty (Figure 2) to stabilize the annulus and to increase the coaptation height. The Carpentier-Edwards Physio II ring (Edwards Lifesciences, Irvine, CA, USA) was sutured with the annulus by using four 2-0 stitches. Three single stitches are passed at the middle of the posterior mitral annulus. The stitches are then passed through the ring and fixed together (Video S1).

The two lateral stitches were then used to perform a running suture between the annulus and ring on both sides of the valve, up to the commissures. A fourth stitch was used for fixing the anterior annulus with the anterior part of the ring and then fixed with the two lateral running sutures (Figure 2 and Video S1). The saline test was then used to calibrate the chordal size, the level of the coaptation, and then the chordae were fixed. The left atrium was closed, de-airing was done, and CPB was weaned out.

## 3. Results

A total of 29 patients, 13 (44.8%) males, with a median age of 58 years (52-68), were included in the study. All these patients were affected by degenerative mitral valve regurgitation and were successfully treated with mitral valve repair with the use of a robotic system at the University Hospital of Bordeaux from January to September 2021. 

Table 2 shows the pre-operative characteristics of the patients. The median body mass index (BMI) and EUROscore II were 23.9 Kg/m^2^ (22–26) and 0.84 % (0.7–1.1), respectively. Most of the patients (59%) were in NYHA functional class II at the time of operation, and the median left ventricle ejection fraction was 65% (60–67). None of the patients had previously undergone cardiac surgery operation.

All patients were affected by severe mitral regurgitation, including two patients with associated mitral fibroelastoma. In 17 (59%) patients, the valve defects were due to isolated prolapse of the p2 scallop. In 4 (14%) patients, the prolapse was diffused on the posterior leaflet (including p1–p2 and p2–p3), and in the remaining 8 (28%) patients, the regurgitation process involved both posterior and anterior leaflets. The median annular size was 37 mm (35–40).

Table 3 shows the intraoperative details. The concomitant procedures performed include atrial fibrillation ablation in 2 (7%) patients, fibroelastoma resection in 2 (7%) patients, tricuspid valve repair with Kay procedure in 4 (14%) patients, and surgical closure of patent foramen ovale in 4 (14%) patients.

**Figure 3:**
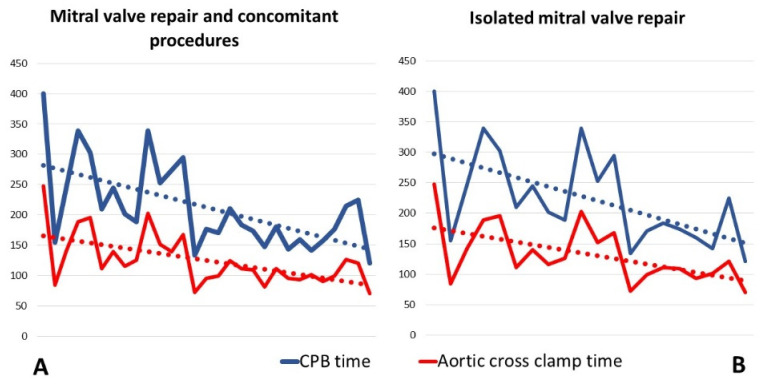
Operative times.These graphics show the progressive reduction of CPB- and cross clamp-time from the beginning or our robotic mitral valve experience. (**A**) Operative times of entire mitral valve repair cohort, including all 29 patients treated with mitral valve repair and concomitant procedures; (**B**) Operative times of isolated mitral valve repair procedures. For this graphic, all patients with mitral valve repair and concomitant procedures were excluded. CPB: cardiopulmonary bypass.

The median cardiopulmonary bypass and aortic cross-clamp times were 189 min (166–250) and 111 min (97–141), respectively, showing a progressive decrease throughout the experience (Figure 3). The median number of implanted neochordae was 2 (2–2), while the median size of the ring used for annuloplasty was 34 mm (32–34). Single stitch suture was added to optimize the valve coaptation in 15 patients, 7 of them on the free margin of the mitral leaflet, and in 8 cases, on the commissural zone. 

The post-operative details are presented in Table 4.

The median ICU and hospital stays were 1 day (1–2) and 8 days (7–10), respectively.

None of the patients reported post-operative bleeding requiring operation, infections, or any pulmonary or kidney injury. One patient experienced a post-operative cardiogenic shock. The coronary angiogram was performed, showed normal coronary arteries without stenosis or injuries. Inotropic support was achieved and kept for 4 days with a favorable clinical course with the progressive recovery of cardiac function.

The in-hospital and 30-day cardiac-related mortality was 0%. In addition, we report the case of one patient dead from suicide during the hospitalization.

The procedure’s success was achieved in 97% of the patients. One patient experienced moderate post-operative mitral regurgitation detected at 5-day post-operative echocardiographic control and due to a too-long neochord. Unfortunately, two months later, the same patient reported a partial rupture of the mitral free margin at the level of the chord implantation, which revealed a severe mitral regurgitation. We opted for a reoperation through the sternotomy approach, which allowed for effective mitral valve repair. The other 28 patients reported a trivial or absent residual mitral valve regurgitation. Until now, 19 (66%) patients completed the 3-months follow-up control, with 17 (59%) having absent/trivial mitral regurgitation and only 2 (7%) patients mild to moderate (2+) residual mitral regurgitation.

The three-month clinical examination reports one case of pleural effusion requiring drainage and no other significant complications. In addition, all patients are in NYHA functional class I.

## 4. Discussion

Mitral valve leaflets surgery during the past decades was undoubtedly marked by professor A. Carpentier who conceptualized the “French correction” for treating failed mitral valve [10,11].

This approach, also called the “resect technique” involving the quadrangular resection of the posterior leaflet, quickly became the gold standard for the treatment of degenerative mitral valve regurgitation, allowing the restoration of the correct valve function. Despite its good results, during the early 2000s, parallel to the resect technique, a new approach was introduced in the surgical practice of mitral valve repair [12]. In fact, the surgical technique was progressively reviewed, and the paradigm of leaflet resection was shifted to the “respect technique”.

The arguments under debate and disputed between the two techniques are basically the surface of the coaptation, the respect of the valve anatomy, and the possible ventricle’s deformation associated with the repair technique. The rationale of the respective technique is to preserve the valve tissue by positioning the expanded polytetrafluoroethylene (e-PTFE) neochordae on the prolapsing leaflet to increase the surface of the coaptation. Another important advantage associated with this technique is the ability to pursue another technique at any time, as nothing is irreversibly removed or altered.

In our series herein presented, we chose to perform the respect technique in all 29 patients, considering this technique safe, reproducible, and effective. Based on our large experience in mitral repair (more than 100 per year at our Institution), we started this journey in robotic mitral repair favoring the respect technique.

This paper focuses on two important considerations that we can make on our initial robotic mitral valve repair experience. The first one concerns a purely technical aspect, and it is related to the running suture technique used for fixing the ring with the mitral annulus. This technique is exclusively used for robotic mitral valve repair at our Institution, while during the mitral repair through the sternotomy approach, separated points are passed on the annulus. The main advantages of this technique are to facilitate the distribution of the stitches evenly, to avoid the passage of too many stitches through the surgical access, and to reduce the annuloplasty times. 

The second important consideration learned from our initial robotic surgery experience concerns the possibility of debunking what, for many surgeons, is a taboo: to start a robotic surgery program without having any previous experience in minimally invasive cardiac surgery. In fact, it is known that robotic surgery represents the last and apparently the most complex step in cardiac surgery, starting from the median sternotomy and passing through the video-assisted mini-thoracotomy. This belief, in addition to the high costs of the procedures, is the cause of the limited diffusion of robotic surgery. However, the most important result of our single-center experience is the progressive reduction of CPB and cross-clamp times after a relatively small sample of operated patients (Figure 3). Indeed, the results achieved after the first 29 patients are comparable to those of centers with consolidated experience in robotic surgery [13,14]. 

As shown in Figure 3, although the beginning of our experience with robotic surgery was inevitably associated with longer CPB and clamp times, subsequent interventions were marked by a rapid learning curve.

Moreover, a great effort was made by the whole team in planning a procedure that was as close as possible to our habits through the sternotomy approach.

The elements that made it possible are the following:

(1) Very careful patient selection;
(2) Dedicated team of anaesthesiologists, perfusionists, nurses, and surgeons;
(3) Expert cardiologist all of the time in the operating room;
(4) The high volume of procedures performed. In fact, the total number of patients treated with robotic surgery was 44 in 9 months (1.2 patients per week). This element certainly played a very important role in reducing the learning curve and increasing the comfort with the DaVinci system.

The main limitation of the study is its retrospective rather than randomized nature. In addition, we are aware that only 29 patients cannot give a solid scientific value to this work. For this reason, further studies with larger samples and longer follow up are needed to demonstrate the efficacy of this technique. In conclusion, our results with the DaVinci system appear to be acceptable and encouraging. A careful patient selection, a dedicated team, and a robust experience in surgical mitral valve repair are the main fundamentals to start a new robotic mitral surgery program.

## Figures and Tables

**Table 1:** The overall population treated with robotic surgery since January 2021 at Bordeaux University hospital.

Variables	Number of Patients (44)
Mitral valve repair	29 (66%)
Mitral valve replacement	3 (7%)
Coronary artery bypass grafting	10 (23%)
Myxoma	2 (5%)
CONCOMITANT PROCEDURES	
Tricuspid valve repair	4
Ablation of atrial fibrillation	2
PFO closure	5

Categorical data are reported as a percentage and absolute frequencies. PFO: patent foramen ovale.

**Table 2:** Pre-operative characteristics.

Variables	Value
Age (years)	58 (52–68)
Gender (male)	13 (45%)
Blood hypertension (n°)	10 (34%)
NYHA functional class	II (II–III)
BMI (Kg/m^2^)	24 (22–26)
Dyslipidemia (n°)	8 (28%)
COPD (n°)	3 (10%)
Smoke (n°)	15 (52%)
Atrial fibrillation (n°)	2 (7%)
LVEF (%)	65 (60–67)
Previous cardiac surgery (n°)	0
EuroSCORE II (%)	0.8 (0.7–1.1)
MITRAL CHARACTERISTICS	
Annular size (mm)	39 (37–40)
Grade of mitral regurgitation	4 (4–4)
Isolated Posterior leaflet prolapse (n°)	20 (69%)
Isolated Anterior leaflet prolapse (n°)	1 (3%)
Bileaflet prolapse (n°)	8 (28%)

Continuous data are reported as median (I, III quartiles); categorical data are reported as a percentage and absolute frequencies. BMI: body mass index; COPD: chronic obstructive pulmonary disease; EuroSCORE: European System for Cardiac Operative Risk Evaluation; LVEF: left ventricle ejection fraction; NYHA: New York Heart Association functional class.

**Table 3:** Intraoperative details.

Variables	Value
CPB time (min)	189 (160–247)
Aortic cross-clamp time (min)	111 (95–140)
Size of implanted prosthetic annular ring (mm)	34 (32–34)
Number of artificial chordae implanted (n°)	2 (2–2)
Commissuroplasty (n°)	8 (28%)
Leaflet reconstruction with patch (n°)	1 (3%)
Cleft closure (n°)	7 (24%)
Concomitant procedure	
Tricuspid valve repair (n°)	4 (14%)
Ablation of atrial fibrillation (n°)	2 (7%)
PFO closure (n°)	5 (17%)
Intraoperative mortality (n°)	0

Continuous data are reported as median (I, III quartiles); categorical data are reported as a percentage and absolute frequencies. CPB: cardiopulmonary bypass, Patent foramen ovale.

**Table 4:** Post-operative characteristics.

Variables	Value
ICU stay (days)	1 (1–2)
Hospital stays (days)	8 (7–10)
Reoperation for bleeding (n°)	0
Conversion to sternotomy (n°)	0
Pulmonary infection (n°)	0
Stoke (n°)	0
Acute kidney injury requiring dialysis (n°)	0
New onset of atrial fibrillation (n°)	0
SAM (n°)	0
Infective endocarditis (n°)	0
30-day overall mortality (n°)	1 (3%)
30-day cardiac related mortality (n°)	0
Postoperative LVEF (%)	55 (52–60)
Post-operative residual mitral regurgitation	
Trivial (n°)	28
Mild (n°)	0
Moderate (n°)	1
Severe (n°)	0

Continuous data are reported as median (I, III quartiles); categorical data are reported as a percentage and absolute frequencies. ICU: intensive care unit; LVEF: left ventricle ejection fraction; SAM: systolic anterior motion.

## Data Availability

The data will be shared on reasonable request to the corresponding author.

## Supplementary Materials

The following are available online at https://www.mdpi.com/article/10.3390/jcm10225439/s1, Video S1: The robotic mitral valve repair.
